# Effects of Heat-Treated *Bifidobacterium longum* CECT-7384 Combined with Fibersol-2 on the Intestinal Health of Cats Submitted to an Abrupt Dietary Change: A Randomized Controlled Study

**DOI:** 10.3390/ani14152179

**Published:** 2024-07-26

**Authors:** Fan Wang, Siyuan Gao, Qianqian Peng, Lili Tan, Siyu Chen, Zhaofei Xia

**Affiliations:** 1 College of Veterinary Medicine, China Agricultural University, Beijing 100193, China; wangfan1112024@163.com (F.W.); gaosiyuan@cau.edu.cn (S.G.); 2 ADM (Shanghai) Management Co., Ltd., Shanghai 200131, China; qianqian.peng@adm.com (Q.P.); lili.tan@adm.com (L.T.)

**Keywords:** abrupt dietary change, *Bifidobacterium longum* CECT-7384, Fibersol-2, fecal microbiota

## Abstract

**Simple Summary:**

Diet significantly influences feline intestinal health, and abrupt dietary changes can disrupt the gastrointestinal balance. Incorporating functional additives, like heat-treated *Bifidobacterium longum* CECT-7384 combined with Fibersol-2, may ameliorate feline gastrointestinal functionality. Heat-treated *Bifidobacterium longum* exhibits anti-inflammatory, antioxidant, and intestinal barrier-enhancing properties. Fibersol-2, widely employed in human foods, enhances colonic mucosal barrier function and increases intestinal microbiota diversity. In this study, we investigated the effects of providing or not providing heat-treated *Bifidobacterium longum* CECT-7384 combined with Fibersol-2 on intestinal barrier integrity, intestinal inflammation, and intestinal microbiota composition in cats undergoing abrupt dietary change. Our study demonstrates that supplementing heat-treated *Bifidobacterium longum* CECT-7384 combined with Fibersol-2 improved the intestinal health of adult cats subjected to abrupt dietary change.

**Abstract:**

Abrupt dietary change can disrupt the intestinal balance in felines. This study aimed to assess the impact of heat-treated *Bifidobacterium longum* CECT-7384 combined with Fibersol-2 on the intestinal health of adult cats before and after dietary change. We selected 24 British shorthair cats, dividing them into two groups. From day 1 to day 14, the control group received a lower protein (33%) concentration (LPF) diet, while the treated group received the same LPF diet supplemented with 0.16% functional additives, consisting of *Bifidobacterium longum* CECT-7384 combined with Fibersol-2. Subsequently, from day 15 to day 28, the control group transitioned to a higher protein (40%) concentration (HPF) diet, while the treated group received the same HPF diet supplemented with 0.16% functional additives. Blood and fresh feces were collected on day 0, 14, 17, 21, and 28 of the experiment. The results suggest that the use of heat-treated *Bifidobacterium longum* CECT-7384 combined with Fibersol-2 may improve gastrointestinal function in cats by reducing serum LPS levels and fecal pH, while increasing fecal sIgA levels. In addition, the functional additive regulates the fecal microbiota and its function, promoting intestinal homeostasis and colonization with beneficial bacteria such as *Blautia*. Furthermore, on day 28, there was a significant difference in fecal microbiota beta diversity between the two groups. In summary, the addition of heat-treated *Bifidobacterium longum* CECT-7384 combined with Fibersol-2 contributes to improving the intestinal health of adult cats affected by abrupt dietary change.

## 1. Introduction

Abrupt change in cat dietary patterns may trigger inflammation and disrupt the intestinal microbiota, especially when cats transition to high-protein diets. Since cats are carnivorous animals, commercial cat foods often contain high levels of protein [1]. Employing a gradual transition approach over a span of seven days when introducing a new cat food regimen has been shown to mitigate the occurrence of gastrointestinal disturbances. Nevertheless, the prevalence of feline diarrhea resulting from dietary change persists, primarily due to challenges in owner compliance and inherent individual variations among cats.

Extensive research has focused on probiotic microorganisms for treating intestinal disorders [2,3]. Furthermore, probiotic microorganisms also show promise in managing metabolic conditions like obesity and diabetes by influencing the composition of the intestinal microbiota [4]. However, against the backdrop of rising antimicrobial resistance, the role of probiotics in alleviating gastrointestinal diseases in pets has been repeatedly highlighted, though their efficacy is often determined by specific strains [5,6]. Additionally, it is imperative to recognize that the safety concerns associated with probiotics should not be underestimated. A study [7] observed that, in patients with predicted severe acute pancreatitis, the probiotic group demonstrated a heightened risk of mortality. Furthermore, probiotic strains can directly cause bacteremia [8]. Therefore, in recent years, some scholars have directed their efforts towards exploring the potential roles of inactivated probiotics, also known as paraprobiotics [9]. *Bifidobacterium* spp. is a widely studied probiotic playing a crucial role in human intestinal health by contributing to various beneficial processes, including enhancement of lactose digestion, manifestation of anti-cancer activity, reduction of serum cholesterol levels, synthesis of B-complex vitamins, and facilitation of calcium absorption [10]. Pasteurized yogurt containing heat-treated *Bifidobacterium longum* has shown mitigative effects on allergic airway inflammation by altering the structure and function of the intestinal microbiota [11]. Additionally, heat-treated *Bifidobacterium longum* exhibits inhibitory effects on proliferation and promotes apoptosis of human colon cancer cells [12]. Compared to probiotics, paraprobiotics not only address the safety concerns associated with the use of live microbial cells but also have an extended shelf life. Currently, there are no studies reporting the effects of heat-treated *Bifidobacterium longum* on the intestinal microbiota and intestinal barrier in cats.

Prebiotics, mainly plant-derived oligosaccharides, resistant starch, and other compounds, serve as fermentation substrates in the gastrointestinal tract. They promote the growth of beneficial microorganisms and contribute to overall intestinal health [13]. Fibersol-2, a resistant maltodextrin (RMD) with prebiotic properties, is a non-viscous, soluble, and fermentable dietary fiber produced from corn starch [13]. Fibersol-2 not only reduces blood glucose, postprandial insulin levels, triglycerides, and serum cholesterol levels, but also promotes intestinal health. It accelerates the transit of feces and stimulates the growth of various beneficial bacteria, indirectly inhibiting the proliferation of potential pathogenic microorganisms [14]. Research indicates that, for adults with insufficient dietary fiber intake, the administration of 25 g of RMD resulted in a 38% increase in the quantity of *Bifidobacteria* in the stool. The stool volume also increased, and participants did not exhibit noticeable gastrointestinal discomfort symptoms [15]. Nathaniel D. Fastinger, PhD and colleagues [16] also observed that the addition of Fibersol-2 significantly increased the abundance of fecal *Bifidobacterium* in a healthy population.

The impact of heat-treated *Bifidobacterium longum* and Fibersol-2 on intestinal health has been extensively studied. However, there is limited research assessing their effects on the intestinal microbiota and immune function in cats. This study marks the first attempt to combine heat-treated *Bifidobacterium longum* CECT-7384 with Fibersol-2 in cats, aiming at exploring their influence on the intestinal health of cats undergoing an abrupt dietary change. It is anticipated that this combination could potentially serve as a dietary management tool to improve feline intestinal health.

## 2. Materials and Methods

### 2.1. Diets

The functional additives (ADM (Shanghai) Management Co., Ltd., Shanghai, China) contain heat-treated *Bifidobacterium longum* CECT-7384 and Fibersol-2. The exact proportions of each component in the formulation are proprietary. The experiment evaluated two different diets with or without the functional additives over two periods. The first diet was a commercial diet for adult cats with a lower protein concentration (33%) (LPF). The second diet was a commercial diet for adult cats with a higher protein concentration (40%) (HPF). The composition and nutritional components of the diets for both groups are detailed in Table 1 and Table 2.

### 2.2. Animals and Groups

**Figure 1:**
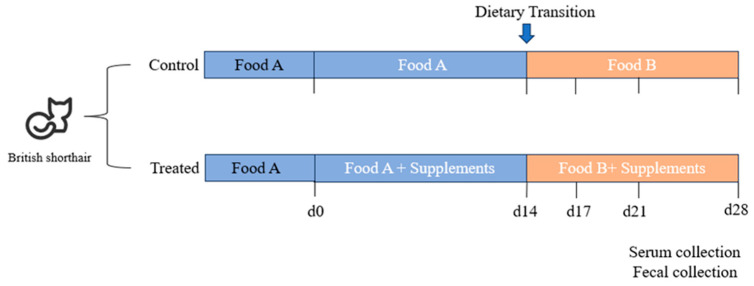
Experimental design and timeline. British shorthair cats were divided into two groups. Body weight data, serum, and feces samples were collected on day 0, day 14, day 17, day 21, and day 28.

Twenty-four British shorthair cats that had not received antibiotics in the past three months and showed no gastrointestinal symptoms were selected from Shanchong Shuifu Pet Nutrition Research Center in Fangshan District, Beijing, China. All cats had been regularly dewormed and immunized, with good overall health. Each cat was housed in a separate cage within the same room. They had free access to food, with fresh food and water provided at 9 a.m. each day, and litter changed at 8 p.m. daily. The cats were divided into two groups, each consisting of 12 cats (6 males and 6 females in each group). The age, weight, and body condition score (BCS) did not exhibit significant differences among the groups (*p* > 0.05). Group allocations are detailed in Table 3. Throughout the experiment, one female cat each from the control and treated groups was excluded: one due to food refusal and the other due to neurological symptoms resulting from an aural polyp. Prior to the commencement of the experiment, all cats were fed an LPF diet for 10 days, designated as the pre-feeding phase. From day 1 to day 14, the control group received the LPF diet, while the treated group received the LPF diet supplemented with 0.16% functional additives. Subsequently, from day 15 to day 28, the control group transitioned to an HPF diet, while the treated group received an HPF diet supplemented with 0.16% functional additives. The experimental design and a timeline of events are shown in Figure 1.

### 2.3. Sample Collection

On days 0, 14, 17, 21, and 28 of the experiment, 2 mL of blood was collected from the medial vein of the hind limbs into non-heparinized blood collection tubes at room temperature. The tubes were allowed to stand for 30 min before being centrifuged at 3000× *g* revolutions per minute for 10 min. The supernatant was then transferred to a 1.5 mL centrifuge tube. Samples were stored at −80 °C in a freezer for future use.

On days 0, 14, 17, 21, and 28 of the experiment, fresh feces were collected, and a portion was extracted. The portion was used for pH and fecal dry matter (DM) evaluation. The remaining feces were placed into a sterile fecal collection tubes and stored at −80 °C in a freezer for future use.

### 2.4. Plasma Parameters and Fecal Parameters Measurement

On days 0, 14, 17, 21, and 28 of the experiment, fresh feces were collected to analyze fecal pH, DM, and fecal score (FS). The Waltham^®^ Fecal Score system (Appendix A) was used, with grades 2–3 indicating good fecal status and grades 3.5 and above indicating excessive fecal moisture. Measurements of pH were conducted using a pH meter (Shenzhen Yage Technology Co., Ltd., Shenzhen, China). Fresh feces (0.5 g) were weighed into a 15 mL centrifuge tube, and 9.5 mL deionized water was added. The sample was centrifuged at 3000× *g* rpm for 5 min after being mixed thoroughly, and the supernatant was poured into a disposable plastic cup. For fresh fecal DM measurement, 5 g fresh feces was dried at 121 °C on a HNB-50T (Xiamen Senbi Technology Co., Ltd., Fujian, China) baking tray.

On days 0, 14, 17, 21, and 28 of the experiment, serum was collected for measurements of lipopolysaccharide (LPS) and diamine oxidase (DAO) using feline-specific commercial ELISA kits (Jiangsu Enzyme-Free Industry Co., Ltd., Taizhou, China). Calprotectin (CALP), secretary immunoglobulin A (sIgA), and myeloperoxidase (MPO) were measured using feline-specific commercial ELISA kits (Jiangsu Enzyme-Free Industry Co., Ltd.)

### 2.5. Microbiome and Functional Analysis

Fecal DNA was extracted using an E.Z.N.A.^®^ soil DNA kit (Omega Engineering, Norwalk, CT, USA) following the manufacturer’s instructions. The quality of the extracted genomic DNA was evaluated by 1% agarose gel electrophoresis. The concentration and purity were assessed using a NanoDrop 2000 microspectrophotometer (Thermo Fisher Scientific, Waltham, MA, USA).

On days 14, 17, and 28, the 16S rRNA gene was amplified with region-specific primers (338F: ACTCCTACGGGAGGCAGCAG and 806R: GGACTACHVGGGTWTCTAAT). The PCR reaction was performed using 25 μL of output from the AxyPrep DNA Gel Purification Kit (AXYGEN, Union City, CA USA), with the following conditions: initial denaturation at 95 °C for 3 min (1 cycle), followed by 30 cycles of denaturation at 95 °C for 30 s, annealing at 55 °C for 30 s, and elongation at 72 °C for 45 s; with a final extension step at 72 °C for 10 min, and holding at 10 °C until halted by the user. Library construction using a TruSeqTM DNA Sample Prep Kit (Illumina, Inc., San Diego, CA, USA) followed by paired-end sequencing using an Illumina Paired-End 300 (PE300) platform (Majorbio, Shanghai, China) were performed according to the standard protocols from the manufacturers.

Bioinformatics analysis of the sequencing data was carried out as follows: the paired-end reads obtained from Illumina sequencing were spliced based on overlap relationships. Sequence quality was controlled and filtered, and the samples were differentiated for Operational Taxonomic Unit (OTU) clustering analysis and species taxonomic analysis.

### 2.6. Calculations and Statistical Analysis

Alpha (α) diversity metrics for the microbiome results were evaluated using the ACE, Chao, Shannon, Simpson, and Sobs indicesby Mothur 1.30.2 software. we employed the Bray–Curtis distance algorithm for beta (β) diversity analysis via Principal Coordinate Analysis (PCoA) and performed differential analysis using Adonis. Relative abundance and differential analyses of community structure were conducted at the phylum and genus levels based on taxonomic information. Due to non-normal distribution, median values (upper quartile, lower quartile) were used. The Scheirer–Ray–Hare test was applied for two-factor analysis of variance. We used Linear Discriminant Analysis Effect Size (LEfSe) to distinguish differential taxa at the genus level and higher taxonomic levels, setting the Linear Discriminant Analysis (LDA) score to greater than 4.0. PICRUSt2 2.2.0 was utilized to predict metabolic pathways at Pathway Level 3.

The main statistical analyses were performed using IBM SPSS 27.0. Two-way ANOVA was used to analyze the differences in intestinal inflammation and intestinal immune indicators between groups. Data conforming to a normal distribution pattern were expressed as mean ± standard deviation, while non-normally distributed data and hierarchical information were expressed as median (upper quartile, lower quartile). Graphing was performed using GraphPad Prism 9.0 software. Statistical significance was defined as 0.01 ≤ *p* < 0.05, highly significant as *p* < 0.01, and a significant trend as 0.05 ≤ *p* < 0.10.

## 3. Results

### 3.1. FS, DM, Fecal pH, LPS, DAO, CALP, MPO, and sIgA

#### 3.1.1. Before Changing the Feed

Table 4 presents the results of FS, DM, fecal pH, LPS, DAO, CALP, MPO, and sIgA. SIgA exhibited no significant differences either within or among the groups. However, it is noteworthy that the longitudinal comparison of fecal pH in the treated group revealed a trend with a *p*-value of 0.05 (*p* < 0.1), suggesting potential differences in cats consuming cat food containing functional additives for 14 days compared to baseline. On the 14th day, serum LPS significantly decreased in the treated group (*p* < 0.01) and showed a significant reduction compared to the control group (*p* < 0.05).

#### 3.1.2. After Changing the Feed

**Figure 2:**
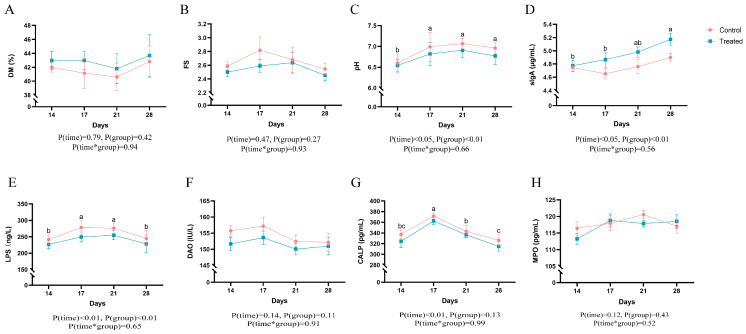
(**A**) DM, (**B**) FS, (**C**) Fecal pH, (**D**) sIgA, (**E**) LPS, (**F**) DAO, (**G**) CALP, (**H**) MPO in cats with or without functional additives; The red line indicates the changes in the parameters for the control group cats, while the green line indicates the changes in the parameters for the treated group cats.; Different superscript letters indicated significant difference (*p* < 0.05), while the same superscript letters indicated no significant difference (*p* > 0.05).

As shown in Figure 2A,B, DM and FS did not differ statistically among days or between the control and treated group. There was an increase in fecal pH after the diet change (Figure 2C). Significant differences in fecal pH are observed on days 17, 21, and 28 compared to the pre-transition period (day 14). On day 28, fecal sIgA in the control group and the treated group was significantly higher than that on day 14 (*p* < 0.05); fecal sIgA in the treated group was significantly higher than that in the control group (*p* < 0.05). Serum LPS levels significantly increased on day 17 and day 21 compared to day 14 but decreased by day 28, showing no statistical difference compared to day 14. Serum DAO levels did not show statistically significant differences among days or between both groups (Figure 2F). Fecal CALP levels did not differ between the control and treated groups, but cats in both groups exhibited a significant increase in CALP values on day 17 compared to day 14 (Figure 2G). Fecal MPO levels did not show statistically significant differences among days or between both groups (Figure 2H).

### 3.2. Fecal Microbiome Composition Profiling

As shown in Table 5, dietary change altered the microbial α diversity indices in both the control and treated groups. Compared to day 14, the richness and diversity of the intestinal microbiota significantly increased on days 17 and 28 (*p* < 0.05). However, there were no significant differences in α diversity indices between the control and treated groups (*p* > 0.05).

**Figure 3:**
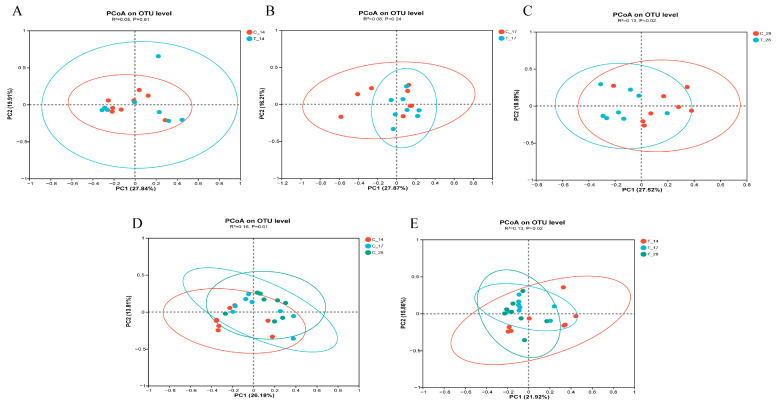
(**A**) PCoA of the composition of the bacterial communities of the control and treated groups on day 14. (**B**) PCoA of the composition of the bacterial communities of the control and treated groups on day 17. (**C**) PCoA of the composition of the bacterial communities of the control and treated groups on day 28. (**D**) PCoA of the composition of the bacterial communities of the control group on day 14, day17 and day 28. (**E**) PCoA of the composition of the bacterial communities of the treated group on day 14, day17 and day 28; C_14, cats of the control group on day 14, C_17, cats of the control group on day 17, C_28, cats of the control group on day 28, T_14, cats of the treated group on day 14, T_17, cats of the treated group on day 17, T_28, cats of the treated group on day 28. Each dot represents an animal. *p*-values between PCoA analyses are from ANOSIM analysis.

The results of the PCoA (Figure 3) analysis indicated significant differences within groups, suggesting a pronounced intervention effect of sudden dietary change on the feline intestinal microbiota. On days 14 and 17, the microbial communities of both the control and treated groups were not fully separated into distinct clusters, but by day 28 they showed clear separation (*p* < 0.05). Most samples from both groups clustered together, except for a few, indicating a significant intervention effect of additive treatment on the feline intestinal microbiota in the later stages of the experiment.

**Figure 4:**
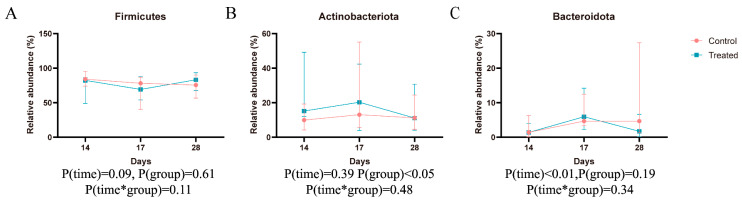
*Firmicutes* (**A**), *Actinobacteria* (**B**), and *Bacteroidetes* (**C**) in both groups on day 14, 17, and 28.

**Figure 5:**
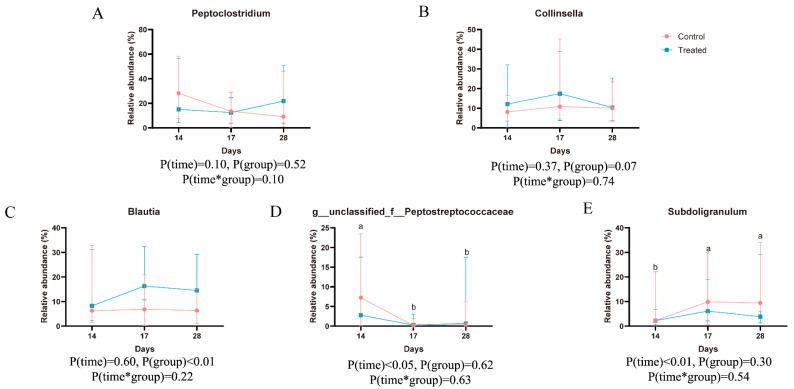
*Peptoclostridium* (**A**), *Collinsella* (**B**), *Blautia* (**C**), *g__unclassified_f__Peptostreptococcaceae* (**D**), and *Subdoligranulum* (**E**) in both groups after the change. ^a,b^ Different superscript letters indicated significant difference (*p* < 0.05), while the same superscript letters indicated no significant difference (*p* > 0.05).

As depicted in Figure 4 and Table 6, at the phylum level there are notable differences in microbial composition both within and between groups. The predominant taxa in each group are primarily concentrated in the phyla *Firmicutes*, *Actinobacteria*, *Bacteroidetes*, *Cyanobacteria*, and *Proteobacteria*. *Firmicutes*, in particular, stands out as the most abundant, with proportions exceeding 60% across all groups, followed by *Actinobacteria.* Following the dietary change, both groups of cats exhibited a notable increase in the relative abundance of *Bacteroidetes* compared to before the change. Moreover, the treated group showed a significantly higher relative abundance of *Actinobacteria* in their feces compared to the control group. At the genus level, ten predominant bacterial taxa were identified in each group (Table 7). Compared to before the dietary change, there was a significant decrease in the relative abundance of *g_unclassified_f_Peptostreptococcaceae* and a notable increase in *Subdoligranulum* in both groups after the change. Additionally, the treated group exhibited a significantly higher relative abundance of *Blautia* in their feces compared to the control (Figure 5).

**Figure 6:**
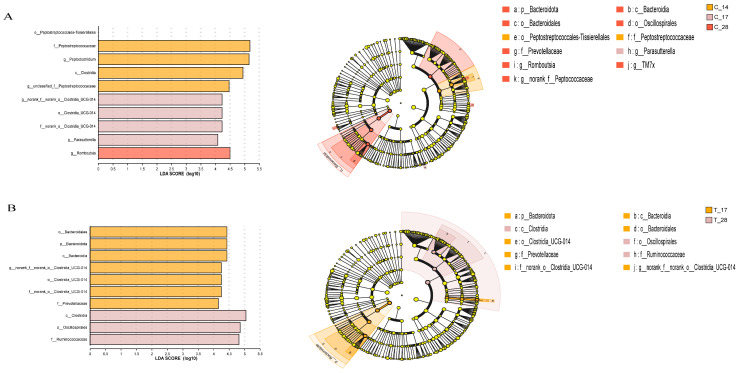
(**A**) Analysis of multilevel species differences of control group on day14, day 17 and day 28; (**B**) Analysis of multilevel species differences of treated group on day 17 and day 28; C_14, cats of the control group on day 14, C_17, cats of the control group on day 17, T_17, cats of the treated group on day 17, C_28, cats of the control group on day 28, T_28, cats of the treated group on day 28.

**Figure 7:**
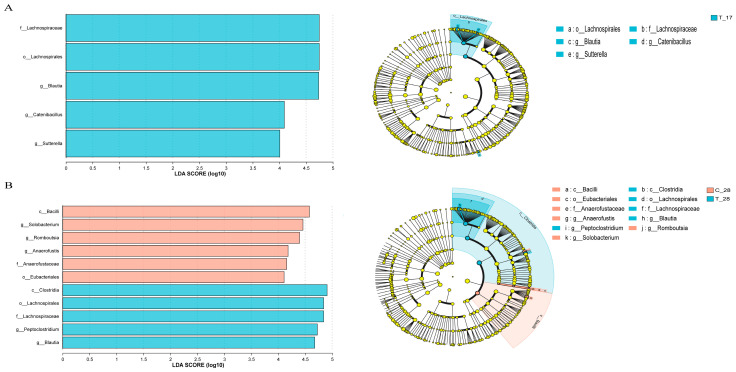
(**A**) Analysis of multilevel species differences between groups on day 17; (**B**) Analysis of multilevel species differences between groups on day 28; T_17, cats of the treated group on day 17, C_28, cats of the control group on day 28, T_28, cats of the treated group on day 28.

To further investigate the differential taxon abundance within and between the two groups, LEfSe was used to compare the bacterial taxa abundance in fecal samples (Figure 6 and Figure 7). In group C_14, *f_Peptostreptococcaceae* and *o_Peptostreptococcales_Tissierellales* were prevalent, and *g_Parasutterella* was enriched in C_17, while *o_Oscillospirales*, *p_Bacteroidota*, *c_Bacteroidia*, *o_Bacteroidales*, *f_Prevotellaceae*, *g_Rombo-utsia*, *g_TM7x*, and *g_norank_f_Peptococcaceae* were observed to be more abundant in C_28 (Figure 6A). *o_Bacteroidales*, *p_Bacteroidota*, *c_Bacteroidia*, *_norank_f_norank_o_Clostridia_UCG-014*, *o_Clostridia_UCG-014*, *f_norank_o_Clostridia_UCG-014*, *f_Prevotellaceae*, and *g_Prevotella* were pronounced in T_17, while *c_Clostridia*, *o_Oscillospirales*, and *f_Ruminococcaceae* were more predominant in T_28 (Figure 6B). On day 17, compared to the control group, the treated group showed higher relative abundance of *f_Lachnospiraceae*, *o_Lachnospirales*, *g_Blautia*, *g_Catenibacillus*, and *g_Sutterella* (Figure 7A). Additionally, no taxa in the control group exhibited LDA values exceeding 4.0. *c_Bacilli*, *g_Solobacterium*, *g_Romboutsia*, *g_Anaerofustis*, and *o_Eubacteriales* were enriched in C_28, while *c_Clostridia*, *o_Lachnospirales*, *f_Lachnospiraceae g_Peptoclostridium*, and *g_Blautia* were significantly more abundant in T_28 (Figure 7B).

### 3.3. Functional Genes

**Figure 8:**
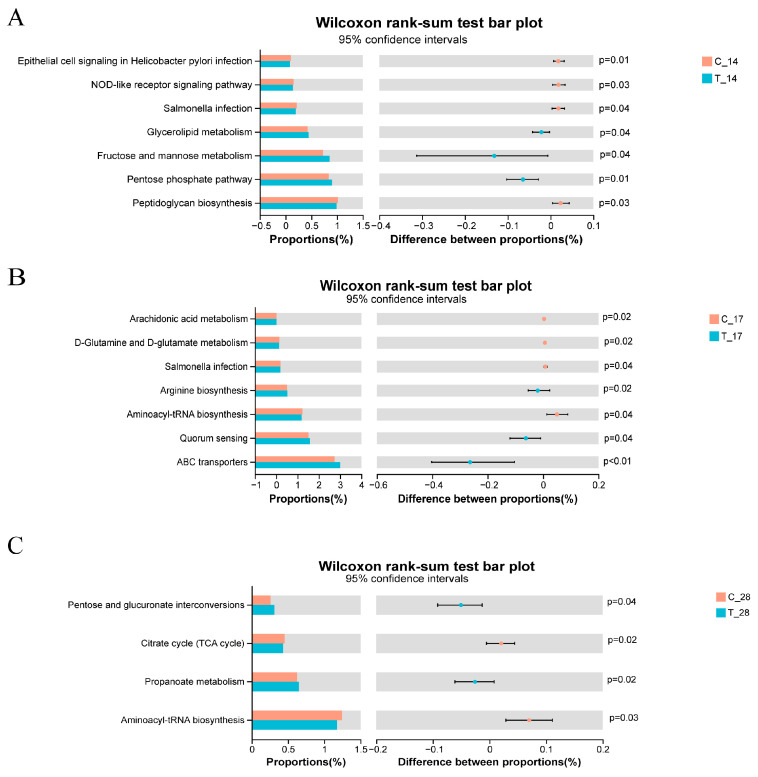
Differential analysis diagram of KEGG level 3 metabolic pathways on (**A**) day 14, (**B**) day 17 and (**C**) day 28; C_14, cats of the control group on day 14, T_14, cats of the treated group on day 14, C_17, cats of the control group on day 17, T_17, cats of the treated group on day 17, C_28, cats of the control group on day 28, T_28, cats of the treated group on day 28.

The presence or absence of functional additives resulted in different expressions of functional genes (*p* < 0.05). As shown in Figure 8, when evaluating only the effects of the diets, an enrichment in epithelial cell signaling in Helicobacter pylori infection, NOD-like receptor signaling pathway, Salmonella infection, and peptidoglycan biosynthesis pathway were observed in the feces of control group on day 14, while the treated group exhibited enrichment mainly in glycerolipid metabolism, fructose and mannose metabolism, pentose phosphate, and the peptidoglycan biosynthesis pathway. On day 17, the control group showed enrichment in pathways related to arachidonic acid metabolism, D-glutamine and D-glutamate metabolism, and aminoacyl-tRNA biosynthesis, whereas the treated group displayed enrichment mainly in the arginine biosynthesis, quorum sensing, and ABC transporters pathways. Furthermore, on day 28, an enrichment in pathways such as the citrate cycle and aminoacyl-tRNA biosynthesis pathways was observed in the control group, while the treated group demonstrated enrichment mainly in the pentose and glucuronate interconversion and propanoate metabolism pathways.

## 4. Discussion

We investigated the effects of heat-treated *Bifidobacterium longum* CECT-7384 combined with Fibersol-2 on gastrointestinal tract stability in adult cats undergoing a diet transition from an LPF diet to an HPF diet. The hypothesis of the study was that the treated group, receiving heat-treated *Bifidobacterium longum* CECT-7384 combined with Fibersol-2, would exhibit a greater ability to adapt to the new diet composition. Our results showed that, before the diet transition, all indices except for LPS were unaffected by supplementation of functional additives. After the abrupt dietary change, all measurements except for pH, LPS, and sIgA concentration remained unchanged, and the functional additives significantly increased the abundance of beneficial bacteria like *Actinobacteria* and *Blautia* in the treated group.

In the present study, the pH values of feces significantly increased in both groups of cats after abrupt dietary change, which is consistent with results observed in humans and dogs [17,18]. This may be attributed to the association between high pH values and protein hydrolysis metabolism [19]. However, Taís Silvino and colleagues [20] reached the opposite conclusion, and the decrease in fecal pH observed in dogs on the HPF diet was accompanied by a decrease in the fecal concentration of short-chain fatty acids (SCFAs). In our study, while both groups showed a rise in fecal pH, the treated group displayed a notably lower fecal pH compared to the control group, possibly explained by the higher levels of *Blautia*. *Blautia*, a beneficial bacterium, produces organic acids such as lactic acid, thereby regulating intestinal pH levels [21].

CALP and MPO are commonly utilized as markers for intestinal inflammation [21,22]. Our findings indicate that MPO did not exhibit statistically significant differences, which may be attributed to its poor stability in feces. Another plausible explanation could be the lack of direct correlation between MPO activity and its expression levels. Selecting assays that assess enzymatic activity rather than relying on available sandwich ELISA might provide a more accurate evaluation of intestinal inflammatory status [22]. In the early stages following dietary change (day 17), both groups of treated animals exhibited a significant increase in fecal CALP levels, suggesting onset of intestinal inflammation due to the dietary change [23], with no differences observed between the groups. Notably, different concentrations of *Bifidobacterium longum* significantly affected CALP measurements [24], indicating that higher additive doses do not necessarily result in better anti-inflammatory effects. This suggests that further experiments are needed to determine the optimal dosage of functional additives for significantly alleviating intestinal inflammation. Consistent with our results, children also showed a significant increase in CALP after short-term RMD treatment [25]. RMD mainly improves animal health by modulating the composition of the gut microbiota. In our experiment, the use of functional additives did improve the composition of the gut microbiota, but this did not include microbes with obvious anti-inflammatory effects, except for *Blautia*, which has some anti-inflammatory properties but seems insufficient to counteract the intestinal inflammation caused by abrupt dietary change [26].

Under normal conditions, LPS remains primarily within the intestine, confined by the intestinal barrier. If this barrier is compromised or permeability increases, LPS may enter the bloodstream, resulting in elevated serum levels [27,28]. During the initial phase of dietary transition, the levels of LPS significantly increased in both groups, possibly due to disruption of intestinal barrier function caused by dietary change [29]. Throughout the entire experiment, the levels of LPS in the treated group were significantly lower than those in the control group, indicating that the additives could significantly reduce the levels of LPS in feline plasma, thereby accelerating restoration of intestinal barrier function impaired by dietary transition. Consistent with our findings, Dong-Yun Lee isolated *Lactobacillus plantarum* NK151 and *Bifidobacterium longum* NK173 from a human fecal bacteria collection, which inhibited Escherichia coli LPS production [30]. Additionally, resistant dextrin has beneficial effects in improving disruption of the intestinal epithelial barrier [31,32]. On the other hand, the ratio of Gram-positive to Gram-negative bacteria in the intestine also influences the detectable levels of LPS [27]. Subsequent analysis of intestinal microbiota revealed a consistent correlation between changes in the abundance of *Bacteroidetes* and variations in LPS concentration. Therefore, we speculate that functional additives may improve intestinal microbiota composition, thereby restoring intestinal mucosal barrier function and suppressing serum LPS levels [33,34].

sIgA stands as a cornerstone within mucosal immunity, acting as a pivotal defense mechanism against infections and playing an indispensable role in protecting the body from pathogen infiltration [35]. Our study revealed that fecal sIgA in the treated group was significantly higher than that in the control group (*p* < 0.05). Consistent with our findings, [36] demonstrates that the intake of grain-derived RS by cats enhances sIgA production. Experiments conducted in human infants [37] and mice [23] also indicate a positive impact of *Bifidobacterium* on sIgA production. In conclusion, cats may benefit from the use of heat-treated *Bifidobacterium longum* in combination with Fibersol-2 to support intestinal immune function.

Upon analyzing the α and β diversity, it becomes evident that, following the dietary change, both groups exhibited a notable increase in the richness and evenness of their fecal microbiota. Moreover, significant disparities in the intestinal microbiota structure were observed, attributed to dietary modification and functional additives, aligning with prior research [19,38]; specifically, a high-protein diet may stimulate microbes to utilize mucin as a nutrient source, thereby increasing the availability of specific substrates such as amino acids, dipeptides, and their metabolites. This process could enhance the diversity and richness of the intestinal microbiota [19].

The phylum *Firmicutes*, representing more than 60% of the bacteria in feline feces, stands as the dominant bacterial phylum, consistent with the findings of previous research [39]. However, contrary to previous studies [40,41], the lower abundance of *Bacteroidetes* observed in this study may be influenced by dietary differences. Following the dietary change, there was a decline in the abundance of the *Firmicutes* phylum, while *Bacteroidetes* exhibited an opposite trend. This shift could be attributed to the potential contribution of a high-protein diet, which may increase the availability of substrates favoring the growth of *Bacteroidetes* over *Firmicutes* [19,42]. However, on day 28, we observed an increasing trend in the relative abundance of *Firmicutes*, which could be explained by the effect of RMD, as indicated by previous research [43,44], Another possible factor is the production of lactate by *Bifidobacterium longum*, which can be utilized by members of *Lachnospiraceae*, thereby stimulating their proliferation [45].

*Actinobacteria* [46], particularly *Bifidobacterium* [47], plays a pivotal role in maintaining intestinal equilibrium and fortifying intestinal barrier function. In this study, *Collinsella*, rather than *Bifidobacterium*, emerged as the dominant genus within the *Actinobacteria* phylum in the feces of cats. In patients with irritable bowel syndrome (IBS), the abundance of *Collinsella* typically decreases [48], while it increases in individuals with improved symptoms, often accompanied by a reduction in pain [49]. In cats, there is evidence suggesting that *Collinsella* is linked to improved fecal scores following a therapeutic response to diet [50]. Although the cats in our study did not show significant diarrhea after the dietary transition, changes in pH, CALP, and LPS levels suggest an imbalance in the intestinal tract post-transition. However, the use of functional additives helped alleviate intestinal disruption.

*Blautia* is a beneficial bacterium that produces organic acids like lactic acid, contributing to intestinal pH regulation and inhibiting harmful bacterial growth. It plays a role in regulating host health and alleviating metabolic syndrome [51]. Previous studies have confirmed a negative correlation between the levels of intestinal *Blautia* and obesity [52,53]. In individuals effectively losing weight, *Blautia* becomes a dominant genus of bacteria [54]. *Blautia* may assist in weight loss by regulating intestinal regulatory T cells and producing SCFAs, thus alleviating obesity-related inflammation [55]. Similarly, analysis of the fecal and mucosal microbial communities in patients diagnosed with inflammatory bowel disease and colorectal cancer consistently indicate a marked decrease in *Blautia* abundance [55,56]. Therefore, the significant increase in *Blautia* abundance observed in this experiment suggests superior intestinal health status in the treated group compared to the control group. It is worth noting that not all inflammatory bowel diseases are negatively correlated with *Blautia* abundance; studies have reported higher levels of *Blautia* in patients with IBS. Due to the fact that patients with IBS are often advised to modulate carbohydrate intake, such as following diets low in fermentable oligosaccharides, disaccharides, monosaccharides, and polyols [57], which typically include indigestible carbohydrates [58], it is likely that the higher levels of *Blautia* observed in patients with IBS are due to *Blautia*’s ability to ferment resistant starch as a substrate [59].

Further analysis using LEfSe indicated an increase in *Parasutterella* abundance in the control group following the dietary transition. *Parasutterella* is associated with the onset and progression of conditions such as IBS and chronic inflammation [60]. Beneficial bacteria [51] such as *Lachnospiraceae*, *Lachnospirales*, and *Blautia* were enriched in the treated group, indicating a possible protective effect of the functional additives against potential pathogenic bacteria and promoting colonization with beneficial bacteria.

In the present study, functional analysis of the intestinal microbiome identified upregulation of genes related to colonization by pathogenic bacteria, such as epithelial cell signaling in Helicobacter pylori infection and Salmonella infection, only in the control group. Salmonella has been verified as a pathogenic factor that contributes to chronic inflammation and carcinogenesis [61]. Helicobacter pylori is implicated in colorectal cancer [62] and gastric carcinogenesis [63]. Moreover, pathways implicated in inflammation, such as arachidonic acid metabolism [64], alongside those pertinent to the establishment of pathogenic bacterial populations, such as D-glutamine and D-glutamate metabolism, exhibited heightened activation [65].

Conversely, in the treated group, the upregulated genes primarily pertained to lipid biosynthesis, carbohydrate metabolism and uptake, and amino acid biosynthesis. On day 28, genes associated with propionate metabolism were enriched in the treated group. Propionate plays a vital role in maintaining intestinal homeostasis by enhancing mucin secretion, strengthening intestinal barrier function, protecting the intestinal mucosa [66], and inhibiting the expression of virulence invasion genes of Salmonella [67]. These genes may contribute to improving gastrointestinal function. However, given the intricate interactions within the gastrointestinal tract, it is imperative to conduct additional metagenomic studies to gain a deeper understanding of these mechanisms.

## 5. Conclusions

Throughout the experiment, abrupt dietary change in cats resulted in intestinal inflammation, compromised intestinal barrier function, and led to dysbiosis. However, the use of heat-treated *Bifidobacterium longum* combined with Fibersol-2 ameliorated gastrointestinal functionality in cats by reducing serum LPS and fecal pH levels, while increasing fecal sIgA levels. Furthermore, the functional additive also regulated the intestinal microbiota and its functionality, fostering intestinal homeostasis and colonization with beneficial bacteria such as *Blautia*. In summary, the addition of heat-treated *Bifidobacterium longum* combined with Fibersol-2 contributes to improving the intestinal health of adult cats affected by abrupt dietary change. However, further studies with longer experimental periods and more replicates are necessary to validate these potential impacts.

## Figures and Tables

**Table 1:** Basic diet composition and nutritional level of the LPF diet.

Diet	Item	Content (%)	Item *	Content (%)
LPF	corn	41.65	CP	33
	chicken oil	8.94	EE	17.2
	fish oil	1	Ash	5.8
	chicken meat powder	36.97	TDF	1.9
	sugar beet pulp	2	Ca	0.9
	calcium hydrogen phosphate	1.24	P	0.9
	calcium carbonate	0.5	Tau	0.2
	sodium chloride	0.2		
	multivitamins and minerals	5		
	chicken hydrolysates	2.5		
	total	100		

* CP: crude protein; EE: ether extract; TDF: total dietary fiber; Ca: calcium; P: phosphorus; Tau: taurine.

**Table 2:** Basic diet composition and nutritional level of the HPF diet.

Diet	Item	Content (%)	Item *	Content (%)
HPF	corn	21.96	CP	40
	chicken oil	8.62	EE	17.4
	soybean meal	15	Ash	12.5
	chicken meat powder	46.72	TDF	2.4
	sodium chloride	0.2	Ca	1.4
	multivitamins and minerals	5	P	1.1
	chicken hydrolysates	2.5	Tau	0.2
	total	100		

* CP: crude protein; EE: ether extract; TDF: total dietary fiber; Ca: calcium; P: phosphorus; Tau: taurine.

**Table 3:** Experimental cat group information.

	N (Female/Male)	Age ^1^ (Month)	Weight ^2^ (kg)	BCS ^3^
Control	12 (6:6)	14 (13, 22.5)	3.44 (3.01, 4.40)	5 (4, 5)
Treated	12 (6:6)	15.5 (13, 33.25)	3.29 (2.97, 3.74)	5 (4.25, 5)
*p*	/	0.38	0.32	0.51

^1^ Age, ^2^ weight, and ^3^ BCS score are expressed as medians (upper quartile, lower quartile); ^3^ BCS, body condition score.

**Table 4:** Comparison of FS, DM, fecal pH, LPS, DAO, CALP, MPO, and sIgA levels between and within groups.

Target	Day	Control	Treated
FS ^1^	Day 0	2.5 (2.5, 2.5)	2.5 (2.5, 2.5)
	Day 14	2.5 (2.5, 2.5)	2.5 (2.5, 2.5)
DM ^2^ (%)	Day 0	41.50 ± 3.65	41.85 ± 5.03
	Day 14	41.98 ± 2.28	42.98 ± 4.22
pH	Day 0	6.63 ± 0.18	6.60 ± 0.12
	Day 14	6.60 ± 0.22	6.54 ± 0.14
LPS ^3^ (ng/L)	Day 0	251.93 ± 12.92	252.62 ± 13.13
	Day 14	241.81 ± 13.97	226.65 ± 13.17 *^,#^
DAO ^4^ (IU/L)	Day 0	153.57 ± 7.77	152.25 ± 8.78
	Day 14	155.73 ± 5.51	151.67 ± 6.79
CALP ^5^ (pg/mL)	Day 0	335.25 ± 22.78	337.37 ± 20.89
	Day 14	337.47 ± 26.53	324.50 ± 39.84
MPO ^6^ (pg/mL)	Day 0	113.01 ± 6.19	111.69 ± 6.60
	Day 14	116.85 ± 6.40	113.23 ± 5.65
sIgA ^7^ (μg/mL)	Day 0	4.70 ± 0.23	4.72 ± 0.75
	Day 14	4.75 ± 0.22	4.78 ± 0.28

^1^ FS: fecal score, ^2^ DM: fecal dry matter, ^3^ LPS: lipopolysaccharide, ^4^ DAO: diamine oxidase, ^5^ CALP: calprotectin, ^6^ MPO: myeloperoxidase, ^7^ sIgA: secretary immunoglobulin A, * compared with the control group, *p* < 0.05; ^#^ means *p* < 0.01 compared with day 0 of the experiment.

**Table 5:** Comparison of α diversity indices.

Index	Group	Day 14	Day 17	Day 28	*p*-Values		
					Time	Time	Time*Group
ACE	C	267.98 ± 86.18 ^b^	312.91 ± 51.18 ^a^	324.38 ± 75.11 ^a^	<0.05	0.69	0.96
	T	244.56 ± 87.86 ^b^	322.73 ± 82.31 ^a^	342.18 ± 100.68 ^a^			
Chao	C	267.88 ± 84.58 ^b^	310.99 ± 54.42 ^a^	329.49 ± 77.30 ^a^	<0.01	0.98	0.67
	T	242.92 ± 87.92 ^b^	321.28 ± 82.77 ^a^	347.03 ± 105.92 ^a^			
Shannon	C	2.61 ± 0.54 ^b^	3.13 ± 0.27 ^a^	3.03 ± 0.43 ^a^	<0.01	0.97	0.57
	T	2.42 ± 0.48 ^b^	3.25 ± 0.40 ^a^	3.08 ± 0.48 ^a^			
Simpson	C	0.16(0.11, 0.26) ^a^	0.10(0.08, 0.11) ^b^	0.10(0.07, 0.17) ^b^	<0.01	0.88	0.41
	T	0.19(0.14, 0.31) ^a^	0.09(0.06, 0.11) ^b^	0.10(0.08, 0.13) ^b^			
Sobs	C	227.00 ± 72.49 ^b^	272.13 ± 44.29 ^a^	273.13 ± 61.89 ^a^	<0.05	0.99	0.7
	T	207.00 ± 73.35 ^b^	277.75 ± 71.72 ^a^	288.50 ± 78.63 ^a^			

^a,b^ Different superscript letters indicated a significant difference (*p* < 0.05), while the same superscript letters indicated no significant difference (*p* > 0.05); data conforming to normal distribution are represented by the mean ± standard deviation, while data not conforming to normal distribution are represented by the median (upper quartile, lower quartile).

**Table 6:** The phyla that have a relative abundance of over 5%.

Phylum Level	Relative Abundance/%	
	C	T
Day 14		
*Firmicutes*	83.99 (80.42, 91.69)	82.25 (72.71, 86.04)
*Actinobacteriota*	9.93 (6.20, 16.93)	15.15 (12.26, 24.38)
*Bacteroidota*	1.41 (0.43, 4.58)	1.38 (1.01, 1.63)
Day 17		
*Firmicutes*	78.08 (74.21, 83.92)	69.11 (61.95, 77.98)
*Actinobacteriota*	13.04 (7.36, 18.90)	20.23 (16.34, 28.66)
*Bacteroidota*	4.67 (4.11, 8.45)	5.92 (3.63, 9.59)
Day 28		
*Firmicutes*	75.46 (58.94, 85.38)	83.18 (75.18, 91.46)
*Actinobacteriota*	11.19 (6.50, 18.71)	11.10 (6.64, 20.42)
*Bacteroidota*	4.67 (3.51, 10.32)	1.71 (1.39, 3.89)

**Table 7:** The genera that have a relative abundance of over 5%.

Genus Level	Relative Abundance/%	
	C	T
Day 14		
*Peptoclostridium*	28.22 (10.61, 48.29)	15.05 (6.38, 48.28)
*Collinsella*	8.16 (5.76, 15.24)	12.12 (10.75, 22.81)
*Blautia*	6.29 (2.65, 26.41)	8.25 (4.44, 15.40)
*g__unclassified_f__Peptostreptococcaceae*	7.23 (0.64, 13.30)	2.77 (0.07, 7.65)
Day 17		
*Peptoclostridium*	13.54 (1.69, 22.06)	12.53 (6.82, 15.50)
*Collinsella*	10.87 (5.64, 16.84)	17.42 (13.76, 23.97)
*Blautia*	6.81 (4.26, 9.13)	16.31 (13.01, 19.77)
*Subdoligranulum*	9.89 (1.24, 21.70)	6.13 (3.92, 13.75)
Day 28		
*Peptoclostridium*	9.12 (2.82, 13.67)	21.86 (15.63, 28.31)
*Collinsella*	10.05 (4.80, 15.43)	10.36 (5.70, 19.11)
*Blautia*	6.34 (3.26, 12.53)	14.52 (8.76, 26.19)
*Subdoligranulum*	9.41 (6.28, 21.76)	3.83 (2.02, 19.72)

## Data Availability

The original contributions presented in the study are included in the article, further inquiries can be directed to the corresponding author.

## Appendix A

**FS**	**Details**
1	Hard dry and crumbly; bullet-like
1.5	Hard and dry
2	Well formed; does not leave a mark when picked up; kickable
2.5	Well formed, with a slightly moist surface, which leaves a mark when picked up; almost sticky to touch
3	Moist, beginning to lose form, leaving a definite mark when picked up
3.5	Very moist, but still has some definite form
4	The majority, if not all the form is lost; poor consistency; viscous
4.5	Diarrhea, with some areas of consistency
5	Watery diarrhea
