# Correction: Wang et al. Effects of Heat-Treated *Bifidobacterium longum* CECT-7347 Combined with Fibersol-2 on the Intestinal Health of Cats Submitted to an Abrupt Dietary Change: A Randomized Controlled Study. *Animals* 2024, *14*, 2179

**DOI:** 10.3390/ani14182668

**Published:** 2024-09-13

**Authors:** Fan Wang, Siyuan Gao, Qianqian Peng, Lili Tan, Siyu Chen, Zhaofei Xia

**Affiliations:** 1 College of Veterinary Medicine, China Agricultural University, Beijing 100193, China; wangfan1112024@163.com (F.W.); gaosiyuan@cau.edu.cn (S.G.); 2 ADM (Shanghai) Management Co., Ltd., Shanghai 200131, China; qianqian.peng@adm.com (Q.P.); lili.tan@adm.com (L.T.)

In the original publication [1], the strain name “*Bifidobacterium longum* CECT-7347” was mistakenly written as “*Bifidobacterium longum* CECT-7384” in the manuscript. 

The following correction has been made to the Title:

“Effects of Heat-Treated *Bifidobacterium longum* CECT-7347 Combined with Fibersol-2 on the Intestinal Health of Cats Submitted to an Abrupt Dietary Change: A Randomized Controlled Study”.

Three corrections have been made to the Simple Summary as follows:

“… Incorporating functional additives, like heat-treated *Bifidobacterium longum* CECT-7347 combined with Fibersol-2, may ameliorate feline gastrointestinal functionality… In this study, we investigated the effects of providing or not providing heat-treated Bifidobacterium longum CECT-7347 combined with Fibersol-2 on intestinal barrier integrity, intestinal inflammation, and intestinal microbiota composition in cats undergoing abrupt dietary change. Our study demonstrates that supplementing heat-treated Bifidobacterium longum CECT-7347 combined with Fibersol-2 improved the intestinal health of adult cats subjected to abrupt dietary change.”

Four corrections have been made to the Abstract as follows:

“Abrupt dietary change can disrupt the intestinal balance in felines. This study aimed to assess the impact of heat-treated *Bifidobacterium longum* CECT-7347 combined with Fibersol-2 on the intestinal health of adult cats before and after dietary change… From day 1 to day 14, the control group received a lower protein (33%) concentration (LPF) diet, while the treated group received the same LPF diet supplemented with 0.16% functional additives, consisting of *Bifidobacterium longum* CECT-7347 combined with Fibersol-2… The results suggest that the use of heat-treated *Bifidobacterium longum* CECT-7347 combined with Fibersol-2 may improve gastrointestinal function in cats by reducing serum LPS levels and fecal pH, while increasing fecal sIgA levels… In summary, the addition of heat-treated *Bifidobacterium longum* CECT-7347 combined with Fibersol-2 contributes to improving the intestinal health of adult cats affected by abrupt dietary change.”

The following correction has been made to the Keywords:

“abrupt dietary change; *Bifidobacterium longum* CECT-7347; Fibersol-2; fecal microbiota”.

The following correction has been made to the Introduction:

“… This study marks the first attempt to combine heat-treated *Bifidobacterium longum* CECT-7347 with Fibersol-2 in cats, aiming at exploring their influence on the intestinal health of cats undergoing an abrupt dietary change…”

A correction has been made to Section 2.1. Diets as follows:

“The functional additives (ADM (Shanghai) Management Co., Ltd., Shanghai, China) contain heat-treated *Bifidobacterium longum* CECT-7347 and Fibersol-2…”

Two corrections have been made to the Discussion as follows:

“We investigated the effects of heat-treated *Bifidobacterium longum* CECT-7347 combined with Fibersol-2 on gastrointestinal tract stability in adult cats undergoing a diet transition from an LPF diet to an HPF diet. The hypothesis of the study was that the treated group, receiving heat-treated *Bifidobacterium longum* CECT-7347 combined with Fibersol-2, would exhibit a greater ability to adapt to the new diet composition…”

The following correction has been made to Citation:

“…Effects of Heat-Treated *Bifidobacterium longum* CECT-7347 Combined with Fibersol-2 on the Intestinal Health of Cats Submitted to an Abrupt Dietary Change: A Randomized Controlled Study…”

The authors state that the scientific conclusions are unaffected. These corrections were approved by the Academic Editor. The original publication has also been updated.
